# Balance Impairment in Patients with COPD

**DOI:** 10.1371/journal.pone.0120573

**Published:** 2015-03-13

**Authors:** Alexandru Florian Crişan, Cristian Oancea, Bogdan Timar, Ovidiu Fira-Mladinescu, Voicu Tudorache

**Affiliations:** 1 Department of Pulmonology, University of Medicine and Pharmacy “Victor Babes”, Timisoara, Romania; 2 Department of Biostatistics and Medical Informatics, University of Medicine and Pharmacy “Victor Babes”, Timisoara, Romania; University of Dundee, UNITED KINGDOM

## Abstract

**Background/Purpose:**

Chronic obstructive pulmonary disease (COPD) is a respiratory disease that results in progressive airflow limitation and respiratory distress. Physiopathological features of COPD suggest that people who suffer from this disease have many risk factors for falls that have been identified in older individuals. The aim of the study was to compare and quantify functional balance between COPD patients and healthy subjects; to investigate the risk of falls in acute stages of the disease and to identify risk factors that could lead to falls.

**Methods:**

We studied 46 patients with moderate-severe COPD (29 stable and 17 in acute exacerbation - AECOPD) and 17 healthy subjects (control group) having similar demographic data. We analyzed the difference in Berg Balance Scale (BBS), Single Leg Stance (SLS) and Timed Up and Go test (TUG) between these three groups and the correlation of these scores with a number of incriminatory factors.

**Results:**

The presence of COPD was associated with significant worsening of balance tests: BBS (55 control, vs. 53 COPD, vs. 44 AECOPD points p<0.001), TUG (8.6 control vs. 12.3 COPD vs. 15.9 AECOPD seconds. p<0.001), SLS (31.1 control vs. 17.7 COPD vs. 7.2 AECOPD seconds p<0.001) which may be associated with an increased risk of falls. Anxiety and depression were significantly associated with decreased balance test scores; anxiety (2 control vs. 6 COPD vs. 9 AECOPD points p<0.001) depression (2 control vs. 7 COPD vs. 12 AECOPD points p<0.001).

**Conclusions:**

According to our results COPD patients in moderate-severe stages and especially those in exacerbation have a high risk of falls.

## Introduction

COPD is a respiratory disease that results in progressive airflow limitation and respiratory distress. In addition to the pulmonary pathology, patients with COPD develop other manifestations of the disease such as cardiovascular comorbidities, peripheral muscle dysfunction, weight loss, systemic inflammation and psychological problems.[1] Decreased exercise capacity, functional mobility and peripheral muscle performance have been well demonstrated in patients with COPD[2] but emerging evidence is showing that these patients have an important deficit in balance control.[3]

The ability to maintain stability and balance is critical for functional independence in activities of daily living, mobility and for avoiding falls. An impaired balance has been associated with an increased risk of falls resulting in a higher mortality rate among older adults.[4]

Falls are an important health problem with significant consequences for older adults. It is estimated that 30–50% of people over 65 years old fall at least once a year.[5] Tinetti et al. defined a fall as “an event which results in a person coming to rest unintentionally on the ground or lower level, not as a result of a major intrinsic event or overwhelming hazard”.[6]

The risk factors for falls can be divided into intrinsic and extrinsic. Intrinsic factors are patient-related and include: chronic diseases, advanced age, gait deviations, muscle weakness, multiple medications and altered mental status. Extrinsic factors include dangerous activities such as walking on slippery surfaces, improper footwear, unstable living conditions or environmental hazards.[4] It is reasonable to assume that the risk of falls increases as the numbers of risk factors accumulate.

Physiopathological features of COPD suggest that people who suffer from this disease have many risk factors that had been also identified in older individuals, such as, muscle weakness, multiple medications, polyneuropathy.[7]

Falls are not only associated with mortality and morbidity but are linked to poorer overall functioning and early admission to long-term care facilities; therefore reducing fall risk is an important public health objective.[8]

Tinetti et al. showed in a community-dwelling of older adults that the risk of falling was 8% in those who had no risk factors and increased up to 78% for those who had four or more risk factors.[6]Falls that do not lead to injury frequently begin a downward spiral of fear that leads to inactivity and decreased agility, strength and balance and often result in loss of independence in normal activities of self-care.[9]

Throughout the years, a number of instruments have been developed to quantitatively measure balance in the elderly population. These screening instruments are used to evaluate the ability to maintain balance and subsequently, to identify those individuals who present a substantial risk of falling in the very near future.[10]

The choice of the balance tests depends on the purpose of the assessment. Searching the literature we found that the most common used tests are: the Berg Balance Scale (BBS), Timed Up and Go (TUG) and single-leg stance test (SLS). Often Activities Balance Confidence scale (ABC) is considered a part of the clinical balance assessment.[11,12,13]

## Study Aim

The aim of this study was to compare and quantify functional balance between patients with COPD (stable and acute phases) and healthy individuals, to investigate the risk of falls in acute stages of the disease and to find which risk factors could be significant predictors for falls in these patients. Based on the premises that these patients have a higher risk of falls than their healthy peers we consider that analyzing the risk of falls in patients with COPD is of a paramount importance.

## Material and Methods

### Study design

Prior to participation in the study, all patients signed an inform consent form, previously approved of the ethical board of the Clinical Hospital of Infectious Disease and Pneumophtisiology “Dr. Victor Babes” Timisoara.

On admission day, blood samples and arterial gases were collected from all COPD patients. Pulmonary functions results were obtained through spirometry. On the same day patients performed all the balance tests and questionnaires.

### Subjects

We included 29 stable COPD patients who have clinically stable airway obstruction and 17 AECOPD patients. (former smokers >10 packs-year) that did not require hospitalization in Intensive Care Unit, having a borderline hypoxemia but who met the criteria according to the international guideline (Table 1).[14,15] An exacerbation was defined as an increase of respiratory symptoms for two consecutive days, with at least one major symptom (dyspnoea, sputum purulence or sputum volume) plus either another major or a minor symptom (wheeze, cold, sore throat, or cough).[16] In addition 17 healthy individuals, with similar baseline characteristics, without COPD and with normal spirometry (FEV_1_ ≥80% predicted, FEV_1_/FVC ≥0.7) and absence of any health related problems that may have impaired balance and mobility volunteered to participate in the study.

**Table 1:** Subject characteristics data.

Parameter	Controls	Stable COPD	AECOPD	p (A vs. B vs. C)
No. pts.	17	29	17	
No. comorbidities	1.94	2.44	2.35	
Age (years)^§^	61.4 ± 4.0	62.2 ± 5.0	63.1 ± 4.6	0.438
BMI (kg/m^2^)^§^	25.3 ± 3.9	25.4 ± 3.6	24.7 ± 5.7	0.867
FVC (%)	95 [15]	73 [13]	58 [10]	<0.001 *
FEV_1_ (%)	95 [18]	29 [7]	28 [7]	<0.001 *
FVC/FEV_1_	79 [9]	33 [10]	36 [13]	<0.001 *
pCO_2_ ^§^	n/a	41.6 ± 6.8	59.2 ± 11.0	<0.001 *
pO_2_ ^§^	n/a	66.1 ± 10.1	61.7 ± 11.0	0.179

* Differences are significant.

^§^ Variables are Gaussian distributed. Values are presented as mean ± standard deviation. p value was calculated using ANOVA test.

^¥^ Variables are not-Gaussian distributed. Values are presented as median and [interquartile range]. p was calculated using Kruskal-Wallis test.

Exclusion criteria included: syncope, inability to communicate, use of medication that may have increased the risk of falls (e.g. antidepressant drugs), postural orthostatic hypotension, neurological or musculoskeletal diseases that could account for possible falls and imbalance, such as Parkinson`s disease, history of cerebrovascular accident, acute or serious cardiovascular problems, transient ischemic attacks or lower-extremity joint replacements.

### Balance measures

Patients first completed the ABC questionnaire that includes 16 items representing basic daily living tasks and more difficult ones performed in the community. Respondents provide ratings on a 0–100% continuous scale based on their confidence related to balance and stability. Before the balance test started blood pressure in supine and standing position was recorded. The BBS was performed after the patients completed the questionnaire. The BBS is a 14 item scale for simple balance tasks and is considered the gold standard test for static and dynamic balance abilities.[17] The degree of success in achieving each task is given a score of 0 (unable) to 4 (independent) and the final measure is the sum of all the scores. Afterwards they performed the TUG which is a test of general mobility, a timed performance measure that includes the balance and gait maneuvers used in everyday life and SLS which is a task of static balance that records the time a participant is able to stand on one leg unassisted. Participants were encouraged to rest as needed throughout the assessment session. The TUG and SLS were performed 3 times with pause between repetitions and the best value was used. The SLS was completed with eyes open first (EO) and then with eyes closed (EC).

To assess the functional exercise level we used the 6 minutes walking distance (6MWD) according to the guidelines of the American Thoracic Society (ATS).[18]

Although there are many risk factors that lead to an impaired balance and falls we assessed the following factors: hypoxemia, anxiety and depression and COPD assessment test (CAT questionnaire) which evaluates the disease impact on the patient.[19]

### Statistical analysis

Data were collected and analyzed using the SPSS v.17 software suite (SPSS Inc. Chicago, IL, USA) and are presented as medians and interquartile range for continuous variables without Gaussian distribution or number of cases and percentage for categorical data.

To assess the significance of the differences between groups, Mann-Whitney-U and Kruskal-Wallis tests (medians, non-Gaussian populations) respectively chi-square for trend (percentages, categorical variables) were used. Continuous variable distributions were tested for normality using Shapiro-Wilk test. The correlation between studied variables was evaluated using Spearman’s rank sum correlation coefficient (non-Gaussian distributed variables), it’s statistical significance being assessed using the t-distribution score test. In this study a p value of <0.05 was considered as the threshold for statistical significance.

## Results

In our study group, the presence of COPD was associated with increased risk of falls, quantified using the parameters of different balance tests. The AECOPD patients had a further increased risk of falls. We observed significant worsening of all the balance tests parameters (Table 2), with the exception of BBS and 6 MWD between controls and stable COPD group. Also, the anxiety and depression scores were significantly higher in the AECOPD group compared to stable COPD vs. control.

**Table 2:** Balance parameters, anxiety and depression in the three studied groups.

	A	B	C			
Parameter	Controls	Stable COPD	AECOPD	p (A vs. B vs. C) ^§^	p (B vs. C) ^¥^	p (A vs. B) ^¥^
SLS EC (sec)	8.5 [6.6]	3.9 [3.4]	1.5 [2.9]	<0.001 *	<0.001 *	<0.001 *
SLS EO (sec)	31.1 [15.6]	17.7 [8.4]	7.2] 4.4]	<0.001 *	<0.001 *	<0.001 *
ABC (%)	97.9 [3.8]	74.6 [14.8]	45.5 [35.8]	<0.001 *	0.001 *	<0.001 *
TUG (sec)	8.6 [2.5]	12.3 [2.1]	15.9 [5.8]	<0.001 *	0.002 *	<0.001 *
BBS (points)	55 [2]	53 [7]	44 [6]	<0.001 *	<0.001 *	0.445
6 MWD	521 [92]	412 [120]	214 [187]	<0.001 *	<0.001 *	0.032 *
CAT	3 [6]	16 [8]	30 [10]	<0.001 *	<0.001 *	<0.001 *
Anxiety score	2 [3]	6 [4]	9 [5]	<0.001 *	0.002 *	<0.001 *
Depression score	2 [2]	7 [5]	12 [3]	<0.001 *	<0.001 *	0.008 *

Variables are not-Gaussian distributed. Values are presented as median and [interquartile range].

* Differences are significant.

^§^ p value was calculated using Kruskal-Wallis test.

^¥^ p value was calculated using Mann-Whitney U test.

A series of significant correlations were found between inflammatory markers and the items measured in the balance tests. The inflammation markers are represented by erythrocyte sedimentation rate (ESR), C-reactive protein (CRP) and fibrinogen.

While ABC, TUG, 6 MWD, and CAT significantly reverse correlated with all of these markers, SLS and BBS were reverse correlated significantly only with ESR value (Table 3).

**Table 3:** Correlations between balance tests and inflammatory markers.

Parameter	ESR	Fibrinogen	C-reactive protein
SLS EC	-0.234	-0.183	-0.86
SLS EO	-0.296 *	-0.270	-0.214
ABC	-0.294 *	-0.303 *	-0.315 *
TUG	0.351 *	0.494 **	0.455 **
BBS	-0.336 *	-0.034	-0.106
6 MWD	-0.550 **	-0.432 **	-0.494 **
CAT	0.574 **	0.308 *	0.422 **

Correlations strengths were assessed using Spearman’s rank sum correlation coefficient.

* Correlation is significant at α<0.05 level.

** Correlation is significant at α<0.01 level.

Significant correlations were found also between anxiety and depression scores on one hand and all the studied inflammatory markers and balance parameters on the other hand (Table 4).

**Table 4:** Correlations between depression and anxiety and balance parameters and inflammatory markers.

Parameter	Anxiety	Depression
SLS EC	-0.364 *	-0.419 **
SLS EO	-0.586 **	-0.591 **
ABC	-0.690 **	-0.646 **
TUG	0.722 **	0.509 **
BBS	-0.500 **	-0.473 **
6 MWD	-0.608 **	-0.718 **
CAT	0.609 **	0.708 **
ESR	0.450 **	0.390 **
Fibrinogen	0.338 *	0.305 *
C-reactive protein	0.418 **	0.313 *

Correlations strengths were assessed using Spearman’s rank sum correlation coefficient.

* Correlation is significant at α<0.05 level.

** Correlation is significant at α<0.01 level.

**Fig 1:**
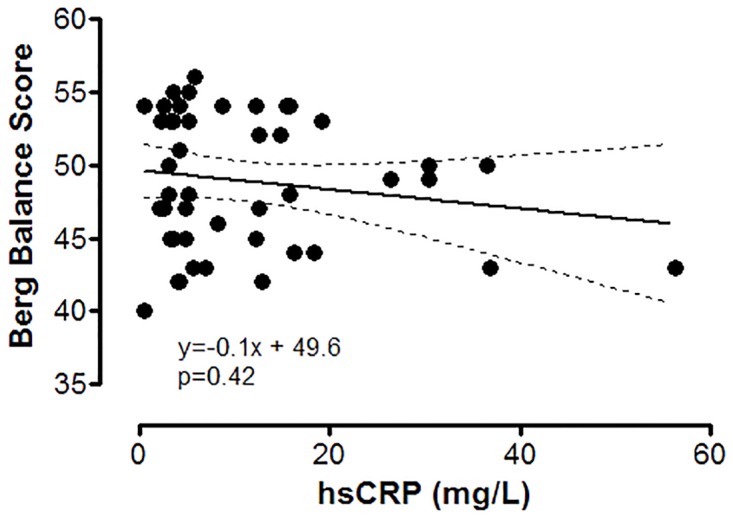
Correlations between hsCRP values and Berg Balance Score in patients with COPD. Interrupted lines are representing the 95% confidence interval for the regression line.

**Fig 2:**
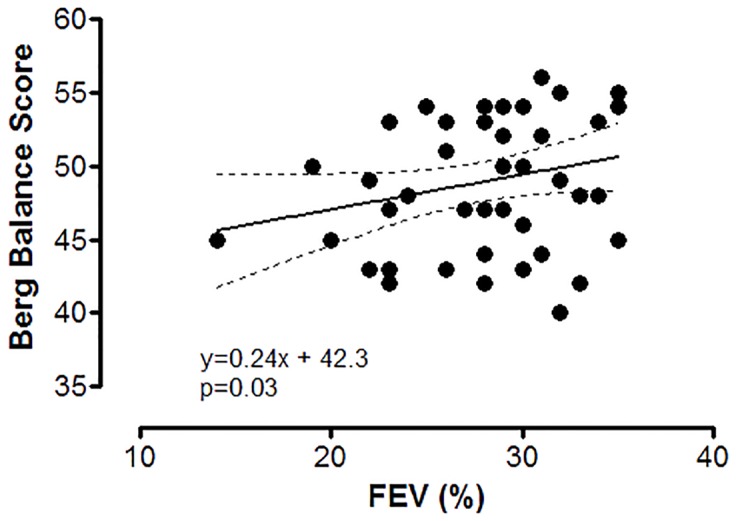
Correlations between FEV (%) and Berg Balance Score in patients with COPD. FEV (%)—forced expiratory volume, percent from expected. Interrupted lines are representing the 95% confidence interval for regression line.

We noted the existence of a negative correlation between the high sensitivity (hs)CRP values and BBS (Fig. 1), respectively a positive, significant one between force expiratory volume (FEV_1_%) and BBS (Fig. 2).

The AECOPD group presented more falls in the previous year based on self reported data. Significant worsening of all the studied balance parameters were observed along with increases in the number of yearly falls (Table 5).

**Table 5:** Comparison of parameters in relation with the number of falls.

	Number of falls in the previous year			p
Parameter	0	1	2	
Stable COPD (%)^§^	18 (62.1%)	11 (37.9%)	0 (0%)	0.001
AE COPD (%)^§^	4 (23.5%)	7 (41.2%)	6 (35.3%)	
SLS EC ^¥^	3.3 [4.1]	3.3 [3.3]	0.7 [2.8]	0.032
SLS EO ^¥^	15.9 [7.1]	13.4 [14.2]	6.4 [5.8]	0.005
ABC ^¥^	74.4 [13.7]	66.6 [31.9]	36.8 [33.4]	0.006
TUG ^¥^	12.0 [2.7]	13.3 [2.9]	17.4 [6.8]	0.002
BBS ^¥^	52.5 [7]	48.5 [10]	44.5 [6]	0.026
6 MWD ^¥^	438.5 [105]	327.5 [179]	157 [167]	<0.001
CAT ^¥^	15.5 [10]	23 [13]	33 [6]	<0.001
Anxiety score ^¥^	6 [3]	8 [6]	9.5 [9]	0.024
Depression score ^¥^	7 [5]	12 [6]	12 [2]	0.005

Note: the number of falls was only assessed for patients with stable and AECOPD, and not for controls.

^§^ Data is presented as number of individuals and (percentage). p value was calculated with chi-square for trend test.

^¥^ Data is presented as median and [interquartile range]. p value was calculated with Kruskal-Wallis test.

## Discussion

This article desires to emphasize the low interest given to the risk of falls in these patients and increase physicians attention to this fact. In this study we have aimed to demonstrate that patients with COPD lose their abilities to maintain balance, especially in acute stages.

Although the fact that individuals with COPD have many risk factors, limited information is available regarding risk of fall and balance in this population.[20] To our knowledge there are no studies that evaluate balance and risk of falls in AECOPD patients. This study assesses balance and risk of falls using 4 types of balance measures together with a functional exercise test on patients in acute stages of the disease.

In a prospective study, Roig et al. estimated that the incidence of falls in COPD patients is 1.2 persons per year, more than four times the incidence reported in the elderly.[21] Another study that assessed whether postural control could discriminate fallers from non-fallers, 46% of the participants reported at least one fall in the previous year.[22] Compared to this study we observed that 37.9% of our stable COPD group had at least one fall during the last year, this number increases to 41.2% in acute stages. Moreover 35.3% of the studied AECOPD patients experienced more than one fall per year. Spruit el al. observed that due to prolonged bed rest caused by an exacerbation, quadriceps peak torque was reduced in COPD patients. Exacerbations have a negative effect on muscle function and physical activity levels and this could be an explanation of the high risk of falls in these patients.[23] Another explanation for this high risk of falls is that COPD exacerbations are frequently associated with deterioration in gas exchange and hypoxemia.[24]

In a study that used BBS, a score of 50 points would present a 10% chance of fall risk, whereas a score of 38 points and lower would represent a 90% chance of falling.[25] Beuchamp et. al. showed that a change of 3 points represents a clinically important difference for this measure.[22]

Compared to these studies our stable COPD patients reached the clinically important difference for this test (53 points) whereas the AECOPD patients approached the score of a very high risk of falls (44 points). The results of our study show that declining BBS scores are associated with an increased fall risk. This low BBS score could be explained by the fact that 40% of the patients have limited exercise capacity due to skeletal muscle alterations. Factors that are found in patients with COPD such as hypoxemia, oxidative stress and systemic inflammation may cause muscle atrophy which is an important risk factor for falls.[26]

The TUG test is often used to screen individuals at increased risk of falls. The most used cut-off score to identify individuals at high risk of falls is ≥ 13.5 seconds. A score above 13.5 seconds indicates a high risk of falls.[27] In our study we observed that the stable COPD group was at the borderline for an increased risk for falls, while the patients in acute stages exceeded the cut-off score (15.9 sec.). Therefore we conclude that the worsening of the symptoms lead to a high chance of falling. Muscle fatigue, which can be defined as the inability of a muscle to maintain a certain force or power output, is a major symptom in COPD patients. An increased load and oxygen need of the respiratory muscles during COPD and reduced venous return compete with an impaired delivery of oxygen to the limb muscles. This could be a reason why our studied patients performed this test so slowly.[28]

In a descriptive meta-analysis, Bohannon et al. presented the appropriate norms for SLS. Individuals with ages between 60–69 have a mean score of 27 seconds with limits of 95%.[29]

Our study outlines the high risk of falls, patients in exacerbation being able to stand on one leg for only 7.2 seconds (median value), and this test being almost impossible to perform with eyes closed (observing a median of only 1.5 seconds in this group).

The 6MWD provides a good estimate of aerobic/endurance capacity and overall functional performance.[30] A significant reduced walking distance was observed between stable *vs*. AECOPD patients (a median value 412 *vs*. 214 meters). We found an association between reduced walking distance and increased fall risk. The 6MWD could be a useful tool to assess the risk of falls in patients with COPD but further work is necessary to determine an appropriate threshold to predict falls in these patients. The explanation for these results is that COPD patients have a peripheral muscle dysfunction (i.e. early onset of lactic acidosis) that appears to be involved in early exercise termination. These individuals have reduced quadriceps endurance thus they cannot perform on the same activity level as their healthy peers.[31] Given this association, we can assume that shorter walking distance can be linked to an increased fall risk.

Fear of falling has been frequently identified as a risk factor for falls. It is reasonable to suppose that reduced balance confidence measured with ABC can be associated with an increased risk of falls.[22] Established normative data from older adults found a threshold of 80 points for the ABC score below which functional impairment starts to decline.[32] We observed that both COPD groups had a decreased ABC score. Lajoie et al. found that a score of 67% is a reliable mean to predict future falls.[25] Our results are consistent with the findings of Beauchamp who compared fallers with non-fallers, thereby we can assume that once a patients has fallen, the confidence in his walking and standing abilities is reduced[22]

Impaired activities of daily living (ADL), which are normally accompanied by poor mobility and reduced physical performance, have been associated with an increased risk for falling in older adults.[33] Julia GS et.al. found a correlation between the CAT questionnaire score and the ADL score. The activities involved in the ADL are: personal care, household chores, leisure and physical activities. Individuals with COPD avoid activities related to gait due to the sensation of dyspnea.[34]

We observed that COPD patients, especially in acute stages have a high CAT score thus having a worse health status. According to Julia GS et al. we can assume that a higher CAT score leads to a more impaired ADL. In this study we found a positive and significant association between the number of falls in previous year and CAT score (p<0.001), demonstrating that an increased CAT score is a valid predictor for falls in these patients.

COPD is associated with depression and anxiety. Mikkelsen et al. reported a prevalence of depression and anxiety in COPD patients between 2–57%.[35] Biderman et. al observed that depression is associated with the number of falls in older adults. Although depression and falls share common risk factors (e.g., impaired ADLs), the underlying mechanism that leads to falls is unclear.[36] Compared to healthy individuals, this high rate of depression could suggest that COPD patients have an increased risk of falls. Our study demonstrated that the patients who suffer from depression (especially the AECOPD patients) have a higher risk of falls. A significant association between the presence of depression and the number of falls in the previous year was found (p = 0.005). One explanation for the risk of falls might be the use of antidepressant medications that can have harmful effects on the physical status of people with depression. Other authors have suggested that depression can result in inattention to potential environmental hazards.[37] Previous studies have found that fear of falling is independently associated with poor mental health and those who report being very afraid of falling have the highest levels of depression. Our results along with those of previous studies, suggest that fear of falling can be a part of a more complex psychological disorder.[38,39] Future studies are needed to determine the exact causation of this phenomenon.

Exacerbations often require hospitalization, pharmacological interventions and prolonged bed rest. Due to the negative effects of exacerbations on physical activity levels and muscle function, gait and balance deficits cannot be missed; we presume that an increased risk for falls secondary to exacerbations is possible. In our study we observed that progression of COPD to AECOPD was associated with an increase trend in the number of falls in the previous year (p<0.001).

Balance and mobility are important elements of most of ADLs and recent studies have shown that hypoxia can have an impairing effect on dynamic and static balance in patients with COPD.[40] Although our COPD patients presented only a borderline hypoxemia even in acute stages, there are multiple factors that increase the risk of falls.[41] We observed that in this study hypoxemia did not play such an important role. Further research is necessary to highlight which factor plays the most important role in falls.

## Conclusions

Our study demonstrates that COPD patients especially in acute stages have an impaired balance and a high risk of falls. Furthermore, the presence of inflammation was significantly associated with worsening in several balance tests, thus being a possible valid predictor for balance impairment in patients with COPD.

## Supporting Information

S1 Dataset (XLS)
